# Ecological and Plant Community Implication on Essential Oils Composition in Useful Wild Officinal Species: A Pilot Case Study in Apulia (Italy)

**DOI:** 10.3390/plants10030574

**Published:** 2021-03-18

**Authors:** Enrico V. Perrino, Francesca Valerio, Ahmed Gannouchi, Antonio Trani, Giuseppe Mezzapesa

**Affiliations:** 1 CIHEAM, Mediterranean Agronomic Institute of Bari, Via Ceglie 9, 70010 Valenzano, Italy; ahmedghannouchi01@yahoo.com (A.G.); trani@iamb.it (A.T.); mezzapesa@iamb.it (G.M.); 2 Institute of Sciences of Food Production (ISPA), National Research Council, Via Amendola 122/O, 70126 Bari, Italy; francesca.valerio@ispa.cnr.it

**Keywords:** correlation, ecology, essential oils, lamiaceae, vegetation

## Abstract

The study focused on the effects of ecology (plant communities and topographical data) on composition of essential oils (EOs) of some officinal wild plant species (Lamiales): *Clinopodium suaveolens, Salvia fruticosa* subsp. *thomasii*, *Satureja montana* subsp. *montana,* and *Thymbra capitata*, in different environments of Apulia (Italy). *C. suaveolens* and *S. fruticosa* subsp. *thomasii* are rare species of conservation interest, while *S. montana* subsp. *montana* and *T. capitata*, have a wide distribution and are used in traditional medicine or as spices. Results showed that the ecological context (phytosociological and ecological features) may influence the composition of EOs of the studied species. High differences in the compound composition have been found in *S. montana* subsp. *montana*, whereas minor effects were observed in *C. suaveolens*, *S. fruticosa* subsp. *thomasii*, and *T. capitata* accessions. The understanding of such aspects is necessary for providing optimal conditions to produce EOs rich in compounds known for their biological activities. The results are of great interest also for EOs producers and at the same time to improve our knowledge and valorize wild officinal plants.

## 1. Introduction

For thousands of years, humans used natural ecosystems for the purpose of their survival [1]. Wild medicinal and aromatic plants were and still are a great resource for the daily life of populations [2,3]. They were and are used as food, spices, cosmetics, and perfumes in religious rituals and to treat illnesses and relieve pain [4]. Hence, historically the use of these wild plants became a significant aspect of populations’ cultural heritage becoming real traditions that kept on from one generation to another [5,6].

Plants, as is well known, are influenced by a range of intrinsic and extrinsic factors, which especially in officinal plants can induce changes in the chemical composition and physiological activities of their essential oils. In fact, it is well documented that the same species, under different environmental and geographical conditions, can produce essential oils with different chemical profiles and biological properties [7,8,9]. However, with the exception of some studies showing a strong correlation between vegetation and heavy metal concentration in soil [10], it seems that there is lack of data on the effect of other important ecological factors, such as plant communities on plant metabolites. Furthermore, despite the great value of some plants as sources of bioactive compounds, important for several applications, many of them are rare and endemic species and have not yet been investigated for their ecology, chemical complexity, and the biological properties of their extracts.

Most of the species belonging to the Lamiaceae family (formerly known as Labiatae) are aromatic and possess antioxidant compounds [10,11] and essential oils useful for defense of the plants against insects [12,13] and making them valuable in cosmetics, perfumery, agriculture, and medicine [14,15]. Indeed, they are widely used in traditional medicine as a cure for many disorders [15], in relation with their global wide distribution, particularly in the Mediterranean region [16], and relative easy propagation. Among the shrub species, they have also high beekeeping importance, at least in the Mediterranean environment, with a few other families such as Ericaceae, Fabaceae (formerly known as Leguminosae), and Rosaceae [17].

The present research focused on four wild taxa, *Thymbra capitata* (L.) Cav., *Salvia fruticosa* Mill. subsp. *thomasii* (Lacaita) Brullo, Guglielmo, Pavone & Terrasi, *Satureja montana* L. subsp. *montana*, and *Clinopodium suaveolens* (Sm.) Kuntze in order to: (1) analyze the essential oils (EOs) extracts from the selected species by GC/MS; (2) assess any relationship between the essential oils composition and the ecological context, especially of plant communities.

## 2. Study Area

**Figure 1:**
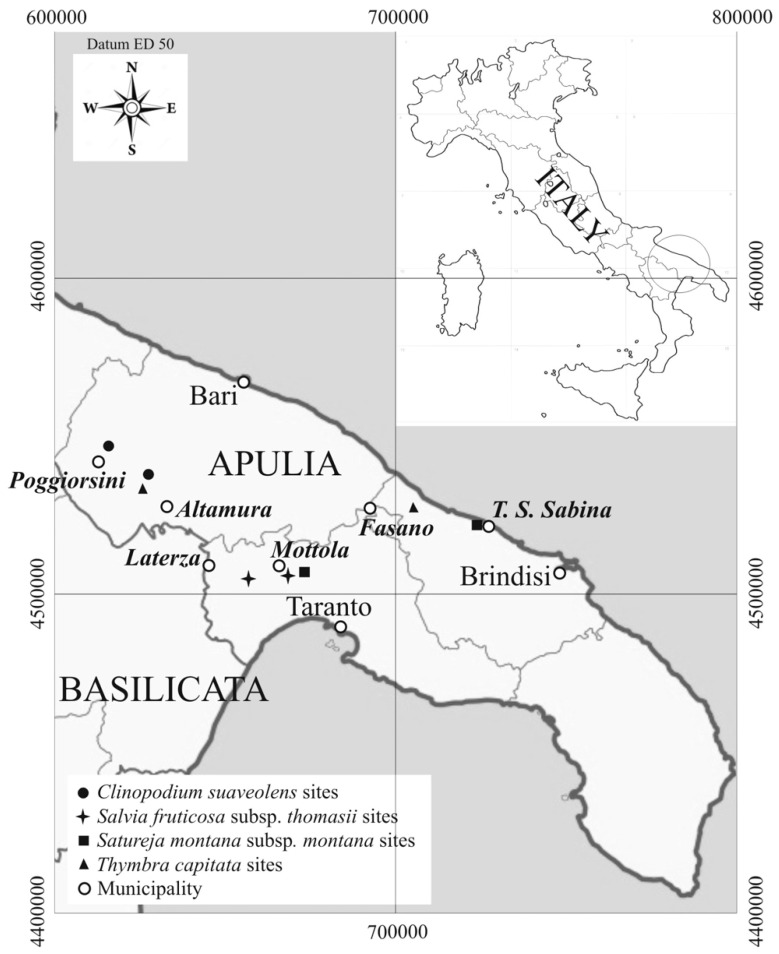
Sites location of the studied officinal wild species.

The studied areas are located in the centre of Apulia (Figure 1), which is one of the most important agricultural areas in Italy. Eight sites, two for each species, were found so distributed: *Salvia fruticosa* subsp. *thomasii* at “Gravine Arco Jonico” (Taranto), near Masseria Gaudella (Laterza, Taranto) and in Gravina del Petruscio (Mottola, Taranto); *Thymbra capitata* at Gravina Capo di Gavito (Mottola, Taranto) and Torre Santa Sabina (Carovigno, Brindisi); *Satureja montana* subsp. *montana* at Difesa di Malta (Fasano, Bari) and Monte Castiglione (Altamura, Bari); Clinopodium suaveolens at Scannapecora (Altamura, Bari) and Jazzo Filieri (Poggiorsini, Bari).

Within the sites in the province of Taranto, the most widespread pedotype is Lithic Ruptic-Inceptic Haploxeralf fine, predominantly clayey, very thin, and very rocky with substrate within 50 cm [18], and in particular at Gravina del Petruscio, Gravina Capo di Gavito, and near Masseria Gaudella, as also at Scannapecora and Jazzo Filieri in the province of Bari, the geological type is that of Skeletal limestones of neritic and carbonate platform facies (Upper Cretaceous) (Geological and Geolithological maps), on Tyrrhenian carbonate reliefs with material defined by calcareous sedimentary rocks and climate from Oceanic to Suboceanic Mediterranean partially mountainous (Ecopedologic map). The Monte Castiglione site shares the same geolithology as the Taranto and Bari sites but with different ecopedology, ascribable to hilly reliefs with undifferentiated tertiary sedimentary rocks and a sub-continental Mediterranean to continental Mediterranean climate (Ecopedologic map). The Torre Santa Sabina and Difesa di Malta sites share the same ecopedology as the Monte Castiglione site (Ecopedologic map) but with Terrigenus-skeletal limestones like “Panchina” (Pleistocene) (Geological and Geolithological maps) (Italian Ministry for the Environment, Land and Sea, http://www.pcn.minambiente.it/viewer, accessed on 18 November 2020) [19].

## 3. Materials and Methods

### 3.1. Vegetation Analysis

The field inspections of the four studied species (*Clinopodium suaveolens*, *Salvia fruticosa* subsp. *thomasii*, *Satureja montana* subsp. *montana*, *Thymbra capitata*) were carried out in 2019. A total of eight vegetation surveys were conducted in two different sites for each species, following the phytosociological method of the Zurich–Montpellier school [20]. Identification code, taxon, location, date, geographic position (expressed in WGS84—World Geodetic System 1984)) are reported in Table 1, while phytosociological and topographical data (Identification code, altitude (m. a. s.), aspect, slope (°), relevé area (m^2^), stoniness (%), rockiness (%), cover total (%), and geolithological and ecopedologic type), endemicity, number of species identified, number of individuals collected for laboratory analyses, are reported in Table 2, Table 3 and Table 4. Other species identified and recorded but not used for the phytosociological classification are here not reported. However, a total of 8 specimens for each species and for each reléve, were collected and deposited at the official herbarium of Bari University (Italy) (*Herbarium Horti Botanici Barensis*, BI). In conclusion, the possible experimental design was 4 species × 2 sites per species x several but variable number of individuals per specimen (Table 2, Table 3 and Table 4).

The identification of the taxa was carried out according to Flora d’Italia [21] and Flora Europea [22], with nomenclature standardized by “An updated checklist of the vascular flora native to Italy” [23] and “An updated checklist of the vascular flora alien to Italy” [24], while the syntaxonomic framework was conceived by several contributions [25,26,27], reported in the phytosociological tables and summarized in the syntaxonomic scheme.

The aerial parts of the studied plant species were harvested on the same sites and dates as the vegetation surveys. The number of individuals collected (from 7 to 150) for the laboratory analysis are reported in the phytosociological tables (Table 2, Table 3 and Table 4).

### 3.2. Laboratory Analysis

The essential oils (EOs) of the selected wild plant species were extracted by hydro-distillation [28] using a Clevenger type apparatus for 4–5 h. Air dried plant material (100 g) was added to 500 mL distilled water in a 1 L volume distillation flask (extraction ratio 1:5 w/vol). EOs were collected in amber glass vials, weighted, and stored at 4 °C. The EOs yield (% *w*/*w*) were determined as grams of EOs per 100 g of dry weight plant material.

The identification of compounds present in the EOs extracts was done by Gas Chromatography coupled with Mass Spectrometry (GC-MS) using a Clarus 680 GC equipped with an Elite-5 MS fused silica capillary column (30 m × 0.25 mm and 0.25 μM film thickness) and interfaced with a single quadrupole mass spectrometer Clarus SQ8C (Perkin Elmer). Mass spectra of target compounds were obtained by an electron impact ionization system with standardized ionization energy of 70 eV. Helium 5.5 was used as a carrier gas at a constant flow rate of 1 mL/min. Mass transfer line and injector temperatures were set at 280 °C and the oven temperature was programmed from 50 °C to 160 °C at 5 °C/min, then raised to 250 °C at 10 °C/min, held at the final temperature for 5 min. Diluted samples (1:10, *v*/*v*, in hexane) were injected in split mode with a split ratio of 1:100. Data were collected in full scan mode in the range 40–300 amu (atomic mass unit). A solvent delay of 4 min was applied. Qualitative results include compound identification and area percentage of related peak in the total ions chromatogram (Table 5).

Compounds identification was performed by both Retention Indexes (RI) [29,30] and mass spectra (MS) search in NIST and Wiley databases (Table 6).

The chemical composition (%) of relative compounds of *Clinopodium suaveolens*, *Salvia fruticosa* subsp. *thomasii*, *Satureja montana* subsp. *montana*, and *Thymbra capitata* is reported in Table 5. Statistical analysis was not applicable due to the low number of samples per species and site, limited amount of material per sample, and few and variable number of individuals per specimen (7–150), due in part also to the fact that the study was pursued on at least two species of conservation interest.

## 4. Results and Discussions

### 4.1. Clinopodium suaveolens (Sm.) Kuntze

**Figure 2:**
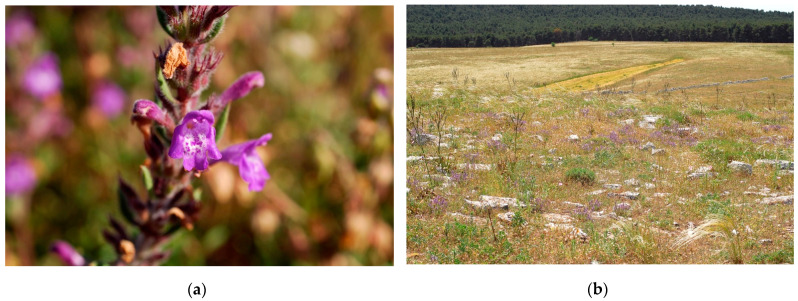
*Clinopodium suaveolens* (Sm.) Kuntze: (**a**) in flowering; (**b**) in its habitat, *Acinos suaveolentis-Stipetum austroitalicae* association.

*C. suaveolens* (synonyms: *Acinos heterophyllus* G. Don, *Acinos suaveolens* (Sm.) Loudon, *Calamintha acinoides* (Ten.) Nyman, *Calamintha acuminata* Friv., *Calamintha langei* Nyman, *Calamintha patavina* Heldr. ex Nyman, *Melissa acinoides* (Ten.) Nyman, *Melissa suaveolens* (Sm.) Nyman, *Satureja suaveolens* (Sm.) Watzl, *Thymus acinoides* Ten., *Thymus melissoides* Bernh. ex Rchb., *Thymus melissoides* Schweigg., *Thymus suaveolens* Sm.) (Figure 2).

Recently, some *Satureja* L., *Micromeria* Bentham, section Pseudomelissa Bentham species and all species of *Calamintha* Miller and *Acinos* Miller were transferred to *Clinopodium* by several authors [31,32,33,34,35,36]. Thus, the number of species belonging to the genus *Clinopodium* has reached about 100. They are mostly distributed in the New World (both temperate and tropical) and temperate Eurasia, but there are a few in Africa, tropical Asia, and Indo-Malaysia [37].

*C. suaveolens* is a Mediterranean species with a north-eastern distribution, reported in Italy, former Yugoslavia, Albania, Greece, Bulgaria, and Turkey [38]. In Italy, it occurs in four Italian regions: Abruzzo, Campania, Apulia, and Basilicata [23], and it is a species of conservation interest cited in the Regional Red List with the status of Vulnerable (VU) for Apulia [39]. It grows with high coverage in the “Alta Murgia” interland (Central part of the Apulia region) in the grassland community of *Acinos suaveolentis-Stipetum austroitalicae* [10,40,41], which is a protected habitat by Directive 92/43 EEC (Eastern sub-Mediterranean dry grasslands (*Scorzoneretalia villosae*) (Code 62A0)). Outside of the Murgian territory, *C. suaveolens* is a typical species of the chamephytic or nanophanerophytic plant communities [42] habitually referred to the *Sarcopoterietalia spinosi* Br.-Bl. 1933 nom. mut. propos Rivas-Martínez, T.E. Díaz, Fernandez-Gonzales, Izco, Loidi, Lousã & Penas 2002 order [43,44].

The data collected about *C. suaveolens*, due to its limited regional distribution and peculiar ecology, as it is one of the characteristic species that gave its name to the *Acinos suaveolentis-Stipetum austroitalicae* endemic association of Alta Murgia (North-West of Apulia), show that it does not grow outside of the just mentioned plant community, with which it shares the same geolithological and ecopedologic context. Consequently, the two detected vegetations are comparable (Table 2).

In the typical context of the *Acinos suaveolentis-Stipetum austroitalicae* community at Scannapecora (Cs1), with five endemic species (*Thymus spinulosus*, *Euphorbia nicaeensis* subsp. *japygica*, *Stipa austroitalica* subsp. *austroitalica*, *Alyssum diffusum* subsp. *garganicum*, *Koeleria splendens*), a high percentage of rockiness (50%), on a slope with 7° of aspect exposed to W-SW, the GC/MS results show the presence of 4 main compounds out of a total of 29 in the EOs. The most abundant component is pulegone with about 80% of the total area, followed by δ-terpineo, D-limonene, and isopulegone (Table 5).

In a more disturbed aspect of the same community at Jazzo Filieri (Cs2), due to an overgrazing explained by the presence of a greater number of species of *Lygeo sparti-Stipetea tenacissimae*, and in particular, a high coverage of *Asphodelus ramosus* L. subsp. *ramosus*, plant not eaten by cattle, less percentage of rockiness (5%), and endemics species, on a slope with 15° of aspect exposed to SW, the EOs composition shows the same composition of Cs1.

The two populations (Cs1 and Cs2) are different in terms of EOs composition percentage, since they show the same compounds but present with different abundance (Table 5). The EOs yield mean for this species was 0.39% with a higher value in Cs2 (+12% of the mean value, *p* < 0.05) in respect to Cs1. Pulegone is the most abundant component in both populations, higher in Cs1 (79.5%) than in Cs2 (75.1%), and therefore the two populations belong to the same pulegone chemotype. One could speculate that at the Jazzo Filieri site (Cs2), the δ-terpineol is the compound that took the place of pulegone at Scannapecora (Cs1) with a 5% increase (from 12.4% to 17.4%), proving to be more suitable for more stressed environments. It is possible to state that this species is partially susceptible to microclimatic, topography, and vegetation changes, which affect the type of EOs, even in close geographycal sites or stations.

It is noteworthy to know that *C. suaveolens* differs from other related *Clinopodium* species as it is strongly odorous, and therefore richer in EOs. Data about EOs of *C. suaveolens* are rather scanty [45,46], due mainly to the natural rarity of the species, though they indicate richness in pulegone as the main phytochemical. Pulegone is a monoterpene ketone [47] often present in the leaf and flower tops of the *Mentha* L. genus. Plant species containing pulegone are used in traditional remedies, flavouring, spices and as an insect repellent [48,49]. They are widely used in Turkey (Balıkesir province) as tea [45], and in Greece as decoction, due to their sedative, diuretic and anti-inflammatory properties [50]. Moreover, the compound exhibits other different biological and pharmacological activities [51], which make it also potentially suitable for an industrial use.

### 4.2. Salvia Fruticosa Mill. subsp. thomasii (Lacaita) Brullo Guglielmo, Pavone & Terrasi

**Figure 3:**
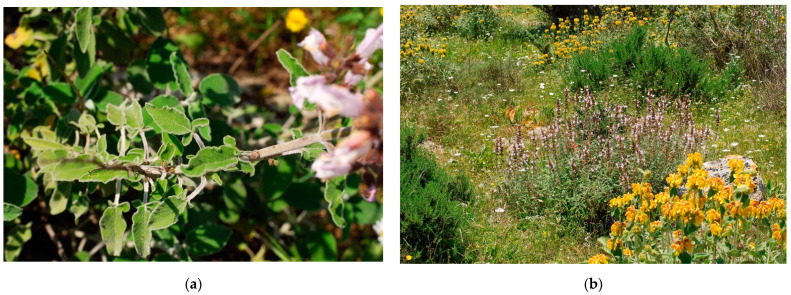
*Salvia fruticosa* Mill. subsp. *thomasii* (Lacaita) Brullo, Guglielmo, Pavone & Terrasi: (**a**) trilobe leaves; (**b**) in its habitat, *Ruto chalepensis-Salvietum trilobae* Biondi & Guerra 2008.

*Salvia fruticosa* subsp. *thomasii* (synonym: *Salvia thomasii* Lacaita, *S. triloba* L.) is one of 1000 species belonging to the *Salvia* L. genus [52]. This perennial shrub is an endemic species located in some regions of the center–south of Italy [23]. In Apulia, the species has been detected in the central-eastern area of the “Gravine Arco Jonico” (Taranto), near Masseria Gaudella (Laterza) and in Gravina del Petruscio (Mottola). Other authors [18,53] observed that Gravina del Petruscio was the richest in individuals and presented a good capacity for renewal, while the population of a few individuals in Masseria Gaudella did not show a similar capacity. In all cases, there are doubts about the ability of this taxon to complete fruit ripening. The only available data are those related to the nominal species, whereas no scientific data related to the specific subspecies exist (Figure 3).

The vegetation of this endemic taxon was detected in Gravina Petruscio (Sf1) and near Gaudella (Sf2) in the province of Taranto. The phytosociological data of both sites (Table 3) only seemingly show comparable topographic conditions within the same plant association *Ruta chalepensis-Salvietum trilobae*, but a careful reading reveals microstational differences of an environmental nature as well as in terms of plant communities, which reflected in the quantity and types of EOs. At Gravina Petruscio in a good environmental condition, in the absence of human disturbances, with 117 m altitude, 35° slope, a low level of stoniness (1%), and a moderate level of rockiness (15%), and 100% coverage of 23 plant species, we observed a richness species of *Oleo sylvestris-Ceratonion siliquae* and *Pistacio lentisci-Rhamnetalia alaterni* communities as *Pistacia lentiscus, Stachys major, Juniperus oxycedrus, Pinus halepensis* subsp. *halepensis, Olea europaea* and *Arisarum vulgare* subsp. *vulgare*, as well as *Salvia fruticosa* subsp. *thomasii* and *Ruta chalepensis*, diagnostic species of *Ruto chalepensis-Salvietum trilobae* association. Some transgressive species of *Cisto cretici-Micromerietea julianae* class, and in particular, *Salvia rosmarinus,* show the natural catenal contact with the two communities.

In less natural condition, with 144 m altitude, greater slope degree (45°), a high level of stoniness (45%) with a low level of rockiness (3%), and medium coverage (40%) of 22 plant species detected, we observed a mosaic community between *Ruto chalepensis–Salvietum trilobae*, without *R. chalepensis*, and *Lygeo sparti–Stipetea tenacissimae*.

These environmental differences are reflected in a higher number of compounds in naturalness conditions of Sf1 with 48 compounds (less disturbed site) in respect to Sf2 with 42 compounds (more disturbed site), with 6 exclusive of Sf1, and 42 of sharing (Table 5). Of particular interest is the presence of borneol, this compound only in Sf1 having a broad spectrum antibacterial activity [54].

In Sf1, the most abundant phytochemicals were eucalyptol (40%), camphor (15%), camphene (6%), α-pinene (5%), and cis-thujone (4%), while the other compounds were almost lower than 3%. In Sf2, the main components are eucalyptol (60%), cis-thujone (5%), α-pinene (4%), α-terpineol (3%), and β-myrcene (4%), while the other constituents do not exceed 2%. Data available in literature on the genus *Salvia* show that eucalyptol (or 1.8 cineole) is usually the most abundant compound and that camphor, α-pinene, β-pinene, and myrcene are common at different concentrations [52,55,56,57]. 

The extraction yield of EOs in *S. fruticosa* showed an average of 0.62% and a higher value in Sf2 (+19% of the mean, *p* < 0.05) than in Sf1.

Eucalyptol (1,8-cineole) is a monocyclic monoterpene found mainly in the *Eucalyptus* genus [58], from which it took its name, and it is a moderately effective antimicrobial agent. Therefore, plants rich in this compound usually rely on the synergetic effect with the other secondary phtyochemicals to exert their potent antimicrobial activity [59].

Finally, data seem to show that in better naturalness condition (Sf1), which meets with its typical vegetation or a high vegetation coverage and plant biodiversity, *S. fruticosa* yields from a moderately to a higher concentration (eucalyptol being an exception) and diversity of chemical compounds than in disturbed conditions (Sf2).

### 4.3. Satureja montana L. subsp. Montana

**Figure 4:**
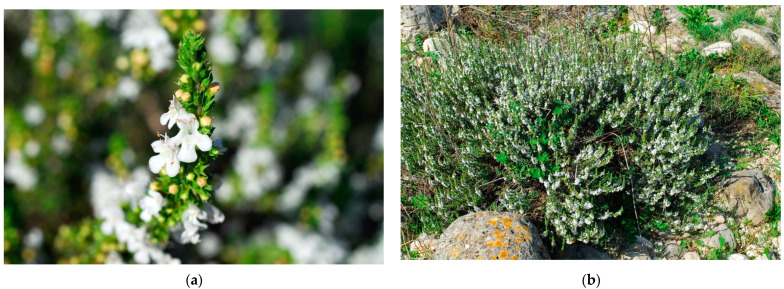
*Satureja montana* L. subsp. *montana*: (**a**) in flowering; (**b**) in its habitat, *Asyneumo limonifolii-Saturejetum montanae*, Biondi & Guerra 2008.

*S. montana* subsp. *montana* is a plant belonging to the *Satureja* L. genus which embraces over 30 species [60]. It is an annual or perennial semi-bushy plant that grows wild in the eastern part of the Mediterranean region [61] and in arid, sunny, stony, and rocky habitats [62]. Also known in Italy as “Santoreggia montana”, this plant has been proven to possess antimicrobial, digestive, laxative, and diuretic properties, making it worthy for cultivation and to be used on an industrial scale [63]. The most abundant components of S. genus extracts are the phenolic phytochemicals thymol and carvacrol [62] (Figure 4).

However, the literature has revealed large variations in the abundance of major components such as γ-terpinene (1 to 31%), p-cymene (3 to 27%), linalool (1 to 62%), and carvacrol (5 to 69%) [64]. These differences are due to the existence of different chemotypes, though differences are also much influenced by the sites, environmental conditions, and the plant vegetative state [62].

The vegetation of this taxon was detected in Difesa di Malta (Sm1) in the province of Brindisi, and Monte Castiglione (Sm2) in the province of Bari. The phytosociological data (Table 4, Sm1) related to the site near the coast (Sm1), at 34 m altitude, with lower rockiness (5%) and stoniness (5%), count 21 species with a covering of 70% of the total area, and must be referred to the *Asyneumo limonifolii-Saturejetum montanae* of the *Festuco-Brometea* class, due to few species of low coverage values with only *Valantia muralis* that has the highest coverage. The few species observed in this syntaxon, often with low coverage values, can be explained by the fact that this plant community grows in an agro-ecosystem, that can defined as an HNFV area (High Nature Value Farmland), within a narrow ecotonal belt, in which different types of vegetation are thickened as is evident from the transgressive species of other syntaxa, such as *Cisto cretici-Micromerietea julianae*, *Quercetea ilicis*, and *Stellarietea mediae*.

In a highly naturalaness environment, in the same ecopedologic context but different geopedology (Table 2 and Table 4), on the eastern side of Monte Castiglione in the Alta Murgia National Park, at 478 m altitude, a garrigue was detected with *S. montana* subsp. *montana* (Table 2, Sm2), to be referred to the *Hyppocrepido glaucae-Stipion austroitalicae* alliance. This vegetation represented the main plant community in the surveyed area, which included also many species referred to the *Lygeo sparti-Stipetea tenacissimae* class, mainly for *Hyparrhenia hirta* subsp. *hirta* and *Asphodelus ramosus* subsp. *ramosus*, both with high coverage. A high coverage of this species suggests that this site has a high grazing load, as this species is not eaten by livestock (mostly sheep). Other different topographic data between the two sites, such as a stronger slope (35°) and rockiness (25%) in the Monte Castiglione site, explain better the differences on their plant communities. Finally, it should be indicated that the phenological stages of sampled species were comparable (no flowering) in both stations due to low temperatures that persisted until the end of April 2019. 

These environmental and vegetational differences have been confirmed by laboratory data, with strong differences in their chemical complexity and abundance of compounds (Table 5).

The *S. montana* subsp. *montana* population coast (Sm1) contained 61 constituents with α-pinene (27%) having the strongest presence, followed respectively by α-terpineol (15.7%), trans-β-ocimene (11%), linalool (7.4%), and D-limonene (7%). Pinene is a major monoterpene usually present in rosemary and lavender. It is known for its insecticidal properties and has two active constituent isomers: α- and β-pinene. The antimicrobial activity of this phytochemical was always under debate with literature indicating the presence of such potential [65,66] and other studies opposing these claims [67,68].

The interland poluplation (Sm2) exhibited 60 compounds. The two populations (Sm1 and Sm2) share 55 compouns, whereas 3 are exclusive of Sm1 and 3 of Sm2. Only α-terpineol, dl-limonene, and trans-β-ocimene with, respectively, 15.2%, 7%, and 11.4% in Sm1 underwent, respectively, a strong reduction to 0.3%, 0.61%, and 1.98% in Sm2, while for other compounds like γ-terpinen the performance was just the opposite: important compounds in the Sm2 become traces in Sm1. Thymol was the most abundant compound in Sm2, representing 46% of its total composition and is almost absent in Sm1. Finally, β-bisabolene, representing 3.5% of the total area in Sm2, was not detected in Sm1.

The EOs yield of *S. montana* subsp. *montana* populations showed the highest observed values during this experimentation with an average of 0.90% and no significative difference between Sm1 and Sm2. 

Terpineol is present in the α form in both Sm1 and Sm2, though it is a monoterpene alcohol commonly used in perfumes and cosmetics and studies have indicated its excellent antimicrobial activity and its ability to alter the morphology of the pathogenic and carcinogenic microorganisms [69,70]. As for β-ocimene, it is present, especially in Sm1, but it is a monoterpene that can play only the role of insect attractants [71] with no notable antimicrobial activities.

### 4.4. Thymbra capitata (L.) Cav.

**Figure 5:**
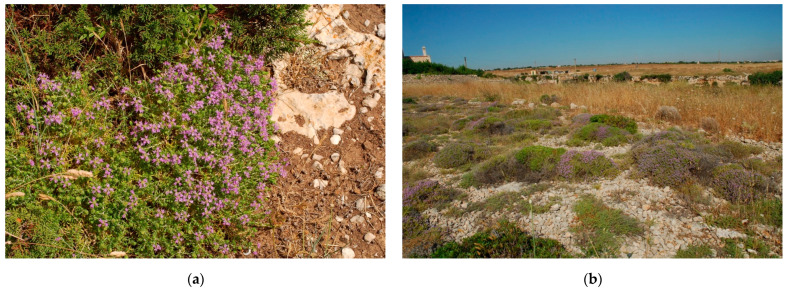
*Thymbra capitata* (L.) Cav. (**a**) in flowering; (**b**) in its maritime habitat.

*T. capitata* (L.) Cav. (synonym: *Thymus capitatus* (L.) Hoffmanns. & Link, and *Coridothymus capitatus* (L.) Rchb. f.) is a perennial shrubby plant that spreads mostly around the eastern Mediterranean coast. It flowers during May and June [72] and occurs in sunny, dry, and rocky places, roadsides, and occasionally on waste grounds or sand dunes [73] at altitudes up to 600 m above sea level [21]. *T. capitata* is the main representative of the genus in the south of Italy [74]. It is one of the most researched officinal species with hundreds of studies analyzing its antimicrobial, antioxidant, anti-inflammatory, and insecticidal properties. These studies have unanimously concluded that the bioactivity of EOs is mainly correlated to the presence of monoterpenes (represented by carvacrol and thymol) that can make up almost 90% of its composition [72,75] (Figure 5).

The vegetation of this taxon was detected at Gravina Capo di Gavito (Tc1) in the province of Taranto (indicated also as Mottola), and Torre Santa Sabina (Tc2) in the province of Brindisi. The Gravina Capo di Gavito site is located at 236 m altitude, a 15° slope and S-SE exposition, with low rockiness (2%), and a slightly higher stoniness (10%). The phytosociological table (Table 4) lists 36 species covering 95% of the surveyed area. The vegetation in which *T. capitata* grows is ascribable to *Asyneumo limonifolii-Saturejetum montanae* association of *Cisto cretici-Micromerietea julianae* class. A high number of species of high coverage were identified, indicating a strong competition with individuals of *T. capitata*. *Phlomis fruticosa*, a diagnostic species of *Cisto cretici-Ericion manipuliflorae* alliance, had the highest coverage, followed by *S. montana* subsp. *montana*. Two transgressive classes were observed, *Lygeo-Stipetea* (perennial grasslands) through several species, such as *Charybdis pancration* and *Hypochaeris achyrophorus*, as well as the *Stellarietea mediae* class.

Torre Santa Sabina is a maritime site with a lower altitude (7 m) than Gravina Capo di Gavito, as well as a lower slope (2°), a NE exposition with equal low rockiness (2%) and stoniness (10%). In this site, *T. capitata* has a higher coverage than Gravina Capo di Gavito and follows the same phytosociological syntaxa. The highest coverage was observed with species referred to the *Cisto cretici–Ericion manipuliflorae* alliance, mainly *Cistus monspeliensis* (3) and *C. creticus* subsp. *eriocephalus* (2). The transgressive *Tuberarietea guttatae * class was observed only in this site.

The relationship between the two sites showed two slightly different conditions within the same class *Cisto cretici-Ericion manipuliflorae*. The association *Asyneumo limonifolii-Saturejetum montanae* was only detected in the relief of Gravina Capo di Gavito due to the presence of a high coverage of *S. montana* subsp. *montana*. The most significant difference between the two sites concern their climatic, geolithological, ecopedologic, and topographical aspects, such as altitudes, continentality (maritime factors in Santa Sabina site), and exposure, that makes the Torre Santa Sabina site cooler than that in the province of Taranto, confirmed also by different transgressive syntaxa observed, except the *Lygeo-Stipetea* class (perennial grasslands, more abundant in Gravina Capo di Gavito), common in both sites.

The higher presence of species of the *Quercetea ilicis* class in the Torre Santa Sabina site seems to suggested a better conservation status of the habitat than the Mottola one, where the observation of *Stellarietea mediae* class species seems to indicate the presence of an anthropic disturbance as witnessed also by the presence of *Centranthus macrosiphon*, an alien naturalized species.

The GC-MS results of Tc1 show the presence of 51 compounds, with the most abundant ones being thymol (31%), carvacrol (26%), γ-terpinene (15.4%), and p-cymene (9.5%). As for Tc2, 50 compounds were detected. The most abundant ones were the same as Tc1 with differences in percentages. Thymol with the strongest presence (36%), followed by γ-terpinene (18%), carvacrol (decreased to 17%), and p-cymene (10%). The rest of the compounds did not exceed 3%. Low differences were observed in the compounds abundance and patterns for the two samples surveyed in different ecological contexts. The two populations shared 50 compounds, while only cis-thujone was exclusive of Tc1. As regards the EOs extraction yield, *T. capitata* had a mean value of 0.54% and Tc1 reported a significative (*p* < 0.05) higher value than Tc2 (+22% of the mean).

Thymol and carvacrol are monoterpene phenols that appear in many EOs of the Lamiaceae family. These compounds have been highly studied and have been proven to possess strong antimicrobial activities against a wide range of microorganisms [76].

Γ-terpinene is a monoterpene, usually present in different plant varieties [77], which can exhibit antioxidant and antimicrobial properties against several microorganisms [78,79]. 

Results for both populations are in agreement with published data; the major compounds of *T. capitata* are usually carvacrol and thymol which are known for their strong biological activities and their amount may be as high as 90% of the total EOs composition [75,80,81,82]. Thus, *T. capitata* may be considered a potential source of antimicrobial phytochemicals.

### 4.5. Syntaxonomical Scheme

See below
*Quercetea ilicis* Br.-Bl. in Br.-Bl., Roussine & Nègre 1952 *Pistacio lentisci-Rhamnetalia alaterni-* Rivas-Martínez 1975  *Oleo sylvestris-Ceratonion siliquae* Br.-Bl- ex- Guinochet & Drouineau 1944   *Ruto chalepensis-Salvietum trilobae-*, Biondi & Guerra 2008*Cisto cretici-Micromerietea julianae*, Oberdorfer 1954- ex Horvatic 1958 *Cisto cretici-Ericetalia manipuliflorae*- Horvatic 1958  *Cisto cretici-Ericion manipuliflorae*- Horvatic 1958   *Asyneumo limonifolii-Saturejetum montanae*- Biondi & Guerra 2008*Festuco-Brometea* Br.-Bl.- et Tx. 1943- ex Klika & Hadac 1944 (habitat 62A0)* *Scorzonero-Chrysopogonetalia*- Horvatic & Horvat (1956) 1958  *Hippocrepido glaucae-Stipion austroitalicae*- Forte et Terzi in Forte, Perrino & Terzi 2005   *Acinos suaveolentis-Stipetum austroitalicae*- Forte & Terzi in Forte, Perrino & Terzi 2005

## 5. Conclusions

Ecological research was conducted in order to know if plant communities’ topographic, geopedological, and ecopedological conditions can induce and/or explain differences in chemical composition of essential oils (EOs). In *Satureja montana* subsp. *montana*, environmental and plant community differences induced significant variations in the abundance of bioactive phytochemicals such as thymol and γ-terpinene. In particular, the EOs from *Salvia fruticosa* had specific differences in their chemical complexity and abundance of components. Passing to *Thymbra capitata*, in Gravina Capo di Gavito (population Tc1), at higher altitude and high plant species coverage, a good production in EOs was observed compared to the coastal population from Torre Santa Sabina (Tc2).

In general, the results showed that there is an interaction, positive or negative, between topographic factors, including plant communities, and yields of EOs. The understanding of such aspects and, more specifically, the knowledge of how and why it happens is very important to design projects aiming to improve valorization and conservation of biodiversity of wild plant species and especially of conservation interest.

The study has provided new data on *Clinopodium suaveolens* and *Salvia fruticosa* subsp. *thomasii*, taxa that from a biochemical point of view seem to be very promising and hence potentially eligible for industrial use, as well as for the enhancement of local genetic resources.

## Figures and Tables

**Table 1:** Identificative code (IC), location, date relief, and geographical position of each taxon.

IC	Taxon	Location	Data Relief	Geographic Position (WGS84)
Cs1	*Clinopodium suaveolens* (Sm.) Kuntze	Scannapecora (Altamura, Bari)	17.04.19	40°54′19″ N 16°26′32″ E
Cs2		Jazzo Filieri (Poggiorsini, Bari)	17.04.19	40°56′37″ N 16°16′23″ E
Sf1	*Salvia fruticosa* Mill. subsp. *thomasii* (Lacaita) Brullo, Guglielmo, Pavone & Terrasi	Gravina Petruscio (Mottola, Taranto)	11.03.19	40°36′34″ N 17°3′53.8″ E
Sf2		Gaudella (Laterza, Taranto)	15.03.19	40°34′28.6″ N 16°50′59.6″ E
Sm1	*Satureja montana* L. subsp. *montana*	Difesa di Malta (Fasano, Brindisi)	22.03.19	40°48′17.7″ N 17°29′53.2″ E
Sm2		Monte Castiglione (Altamura, Bari)	01.04.19	40°52′33.2″ N 16°26′54.6″ E
Tc1	*Thymbra capitata* (L.) Cav.	Gravina Capo di Gavito (Mottola, Taranto)	11.03.19	40°37′47′’N 17°4′5′’E
Tc2		Torre Santa Sabina (Carovigno, Brindisi)	22.03.19	40°45′48.0″ N 17°41′19.6″ E

**Table 2:** *Acino suaveolentis-Stipetum austroitalicae* Forte et Terzi in Forte, Perrino et Terzi 2005.

*Identificative Code*		Cs1	Cs2	Sm2
Altitude (m a. s.)	e n d e m i c	564	512	478
Aspect		W-SW	SW	E
Slope (°)		7	15	35
Relevé area (m^2^)		50	60	40
Stoniness (%)		10	10	10
Rockiness (%)		50	5	25
Cover total (%)		70	85	80
Number of species		41	41	22
Number of individuals collected for laboratory analysis		150	120	50
**Geolithological type**		§	§	§
**Ecopedologic type**		†	†	††
Other species		18	18	3
**Characteristics association**				
***Clinopodium suaveolens* (Sm.) Kuntze**		3	3	-
*Thymus spinulosus* Ten.	E	2	+	-
*Euphorbia nicaeensis* All. subsp. *japygica* (Ten.) Arcang.	E	+	-	+
**Characteristics alliance *Hyppocrepido glaucae–Stipion austroitalicae***				
*Stipa austroitalica* Martinovský subsp. *austroitalica*	E	+	+	3
*Stachys germanica* L. subsp. *salviifolia* (Ten.) Gams		-	+	1
*Alyssum diffusum* Ten. subsp. *garganicum* Španiel, Marhold, N.G.Passal. & Lihová	E	+	-	-
*Linum tommasinii* (Rchb.) Nyman		+	-	-
*Petrorhagia saxifraga* (L.) Link subsp. *gasparrinii* (Guss.) Greuter & Burdet		-	-	1
**Characteristics order *Scorzonero–Chrysopogonetalia***				
*Teucrium capitatum* L. subsp. *capitatum*		1	+	+
***Satureja montana* L. subsp. *montana***		3	-	3
*Carex flacca* Schreb. subsp. *erythrostachys* (Hoppe) Holub		+	+	-
*Euphorbia myrsinites* L. subsp. *myrsinites*		+	+	-
*Anthyllis vulneraria* L. subsp. *rubriflora* (DC.) Arcang.		+	-	-
*Koeleria splendens* C.Presl	E	+	-	-
*Eryngium amethystinum* L.		-	+	-
**Characteristics order *Brometalia erecti* and Cl. *Festuco–Brometea***				
*Festuca circummediterranea* Patzke		1	-	+
*Anacamptis morio* (L.) R.M.Bateman, Pridgeon & M.W.Chase		+	+	-
*Filipendula vulgaris* Moench		+	+	-
*Poterium sanguisorba* L.		-	+	+
*Eryngium campestre* L.		+	-	-
*Ophrys bombyliflora* Link		-	+	-
*Valeriana tuberosa* L.		-	-	+
*Ophrys funerea* Viv.		-	-	+
*Galium corrudifolium* Vill.		-	-	+
**Characteristics/Transgressive class *Lygeo sparti–Stipetea tenacissimae***				
*Asphodelus ramosus* L. subsp. *ramosus*		+	3	3
*Hyparrhenia hirta* (L.) Stapf subsp. *hirta*		-	-	3
*Charybdis pancration* (Steinh.) Speta		-	+	2
*Ferula communis* L. subsp. *communis*		-	2	1
*Reichardia picroides* (L.) Roth		-	+	+
*Clypeola jonthlaspi* L.		-	-	+
*Petrosedum ochroleucum* (Chaix) Niederle		-	-	+
*Thapsia asclepium* L.		-	-	+
*Poa bulbosa* L. subsp. *bulbosa*		+	+	-
*Helianthemum salicifolium* (L.) Mill.		+	+	-
*Hypochaeris achyrophorus* L.		+	1	-
*Plantago bellardii* All. subsp. *bellardii*		+	+	-
*Convolvulus elegantissimus* Mill.		1	-	-
*Valantia muralis* L.		+	-	-
*Brachypodium distachyon* (L.) P.Beauv.		-	1	-
*Dactylis glomerata* L. subsp. *hispanica* (Roth) Nyman		-	+	-
*Thapsia garganica* L. subsp. *garganica*		-	+	-
*Clypeola jonthlaspi* L.		-	-	+
*Petrosedum ochroleucum* (Chaix) Niederle		-	-	+
*Thapsia asclepium* L.		-	-	+
E = Endemic species				

**Geopedology**: §, Skeletal limestones of neritic and carbonate platform facies (Upper Cretaceous); §§, Terrigenus-skeletal limestones like “Panchina” (Pleistocene); **Ecopedology**: †, Tyrrhenian carbonate reliefs with material defined by calcareous sedimentary rocks and climate from Oceanic to Suboceanic Mediterranean partially mountainous; ††, Hilly reliefs with undifferentiated tertiary sedimentary rocks and sub-continental Mediterranean to continental Mediterranean climate.

**Table 3:** *Ruto chalepensis–Salvietum trilobae*, Biondi & Guerra 2008.

*Identificative Code*		Sf1	Sf2
Altitude (m a. s.)	endemic	117	144
Aspect		E	S-SE
Slope (°)		35	45
Relevé area (m^2^)		40	40
Stoniness (%)		1	45
Rockiness (%)		15	3
Cover total (%)		100	40
Number of species		23	22
Number of individuals collected for laboratory analysis		7	10
**Geolithology**		§	§
**Ecopedology**		†	†
Other species		9	6
**Characteristics association**			
***Salvia fruticosa* Mill. subsp. *thomasii* (Lacaita) Brullo, Guglielmo, Pavone & Terrasi**	E	1	1
*Ruta chalepensis* L.		+	-
**Characteristics alliance *Oleo sylvestris–Ceratonion siliquae*, order *Pistacio lentisci-Rhamnetalia alaterni***			
*Pistacia lentiscus* L.		4	2
*Stachys major* (L.) Bartolucci & Peruzzi		+	+
*Juniperus oxycedrus* L.		3	-
*Pinus halepensis* Mill. subsp. *halepensis*		3	-
*Olea europaea* L.		1	-
*Arisarum vulgare* O.Targ.Tozz. subsp. *vulgare*		+	-
**Characteristics class *Quercetea ilicis***			
*Asparagus acutifolius* L.		+	-
*Rhamnus alaternus* L. subsp. *alaternus*		+	-
*Quercus ilex* L. subsp. *ilex*		+	-
*Phillyrea latifolia* L.		-	1
**Transgressive class *Cisto cretici–Micromerietea julianae***			
***Thymbra capitata* (L.) Cav.**		+	+
*Salvia rosmarinus* Schleid.		4	-
*Cistus monspeliensis* L.		+	-
**Transgressive class *Lygeo sparti–Stipetea tenacissimae***			
*Hyparrhenia hirta* (L.) Stapf subsp. *hirta*		-	2
*Reichardia picroides* (L.) Roth		-	1
*Bituminaria bituminosa* (L.) C.H.Stirt.		-	+
*Charybdis pancration* (Steinh.) Speta		-	+
*Clypeola jonthlaspi* L.		-	+
*Ononis reclinata* L.		-	+
*Petrosedum ochroleucum* (Chaix) Niederle		-	+
*Valantia muralis* L.		-	+
E = Endemic species			
**Geopedology**			
§: Skeletal limestones of neritic and carbonate platform facies (Upper Cretaceous)			
**Ecopedology**			
†: Tyrrhenian carbonate reliefs with material defined by calcareous sedimentary rocks and climate from Oceanic to Suboceanic Mediterranean partially mountainous			

**Geopedology**: §, Skeletal limestones of neritic and carbonate platform facies (Upper Cretaceous); **Ecopedology**: †, Tyrrhenian carbonate reliefs with material defined by calcareous sedimentary rocks and climate from Oceanic to Suboceanic Mediterranean partially mountainous.

**Table 4:** *Asyneumo limonifolii–Saturejetum montanae*, Biondi & Guerra 2008.

*Identifacative Code*		Tc1	Tc2	Sm1
Altitude (m a. s.)	e n d e m i c	236	7	34
Aspect		S-SE	NE	E
Slope (°)		15	2	7
Relevé area (m^2^)		45	50	20
Stoniness (%)		10	10	5
Rockiness (%)		2	2	5
Cover total (%)		95	100	70
Number of species		36	28	21
Number of individuals collected for laboratory analysis		30	50	40
**Geolithology**		§	§§	§§
**Ecopedology**		†	††	††
Other species		13	6	10
**Characteristics association**				
***Satureja montana* L. subsp. *montana***		3	-	4
**Characteristics/Transgressive *Cisto cretici–Ericion manipuliflorae*, order *Cisto cretici–Ericetalia manipuliflorae*, class *Cisto cretici–Micromerietea julianae***				
***Thymbra capitata* (L.) Cav.**		3	4	+
*Teucrium capitatum* L. subsp. *capitatum*		+	+	1
*Micromeria graeca* (L.) Benth. ex Rchb.		+	+	+
*Cistus creticus* L. subsp. *eriocephalus* (Viv.) Greuter & Burdet		+	2	-
*Lotus hirsutus* L.		+	1	-
*Phlomis fruticosa* L.		4	-	-
*Cistus monspeliensis* L.		-	3	-
*Salvia rosmarinus* Schleid.		-	+	-
*Helianthemum jonium* Lacaita & Grosser		-	-	1
**Characteristics/Transgressive class *Lygeo sparti–Stipetea tenacissimae* and *Festuco–Brometea***				
*Valantia muralis* L.		+	1	2
*Charybdis pancration* (Steinh.) Speta		2	+	-
*Reichardia picroides* (L.) Roth		+	+	-
*Scorzonera villosa* Scop. subsp. *columnae* (Guss.) Nyman	E	+	+	-
*Daucus carota* L. subsp. *carota*		+	-	1
*Hypochaeris achyrophorus* L.		1	-	-
*Clypeola jonthlaspi* L.		+	-	-
*Festuca circummediterranea* Patzke		+	-	-
*Scorpiurus muricatus* L.		+	-	-
*Anthyllis vulneraria* L. subsp. *rubriflora* (DC.) Arcang.		-	1	-
*Convolvulus lineatus* L.		-	1	-
*Carlina corymbosa* L.		-	+	-
*Daucus carota* L. subsp. *maritimus* (Lam.) Batt.		-	+	-
*Eryngium campestre* L.		-	+	-
**Transgressive class *Stellarietea mediae***				
*Urospermum picroides* (L.) Scop. ex F.W.Schmidt		2	-	-
*Calendula arvensis* (Vaill.) L.		+	-	-
*Erodium malacoides* (L.) L’Hér. subsp. *malacoides*		+	-	-
*Geranium molle* L.		+	-	-
*Stellaria media* (L.) Vill. subsp. *media*		+	-	-
*Vicia hybrida* L.		+	-	-
*Bellardia trixago* (L.) All.		-	+	-
*Vicia sativa* L.		-	+	-
**Transgressive class *Quercetea ilicis***				
*Stachys major* (L.) Bartolucci & Peruzzi		+	+	-
*Olea europaea* L.		-	+	+
*Cytisus infestus* (C.Presl) Guss. subsp. *infestus*		-	1	-
*Pistacia lentiscus* L.		-	+	-
*Arisarum vulgare* O.Targ.Tozz. subsp. *vulgare*		-	-	+
**Transgressive class *Tuberarietea guttatae***				
*Brachypodium distachyon* (L.) P.Beauv.		-	-	2
E = Endemic species				
**Geopedology**				
§, Skeletal limestones of neritic and carbonate platform facies (Upper Cretaceous)				
§§, Terrigenus-skeletal limestones like “Panchina” (Pleistocene)				
**Ecopedology**				
†, Tyrrhenian carbonate reliefs with material defined by calcareous sedimentary rocks and climate from Oceanic to Suboceanic Mediterranean partially mountainous				
††, Hilly reliefs with undifferentiated tertiary sedimentary rocks and sub-continental Mediterranean to continental Mediterranean climate				

E = Endemic species; **Geopedology**: §, Skeletal limestones of neritic and carbonate platform facies (Upper Cretaceous); §§, Terrigenus-skeletal limestones like “Panchina” (Pleistocene); **Ecopedology**: †, Tyrrhenian carbonate reliefs with material defined by calcareous sedimentary rocks and climate from Oceanic to Suboceanic Mediterranean partially mountainous; ††, Hilly reliefs with undifferentiated tertiary sedimentary rocks and sub-continental Mediterranean to continental Mediterranean climate.

**Table 5:** Chemical composition (%) of *Salvia fruticosa* subsp. *thomasii* (Sf), *Thymbra capitata* (Tc), *Satureja montana* subsp. *montana* (Sm), *Clinopodium suaveolens* (Cs).

Compound Name	Sf1	Sf2	Cs1	Cs2	Tc1	Tc2	Sm1	Sm2
Methyl 3(Z)-Hexenyl Ether	0.03							
Salvene, *cis-*	0.06	0.09						
3-Hexen-1-ol, *trans-*	0.05				0.10	0.11	0.07	0.08
trans-Salvene	0.01	0.01						
2-Hexen-1-ol, *trans-*	0.01							
1-Hexanol	0.02	0.01						
2-α-Pinene	0.11	0.16						
Tricyclene	0.24	0.01			0.01	0.02	0.11	1.15
α-Thujene	0.39	0.25	0.03	0.06	1.89	1.50		
α-Pinene, (-)-	5.05	3.58	0.51	0.63	0.94	0.69	26.96	0.69
2,4(10)-thujadien	0.03	0.01			0.02	0.01	0.01	0.02
Camphene	6.32	0.51	0.08	0.10	0.27	0.30	0.51	0.18
Verbenene	0.02	0.01					0.21	
Sabinene			0.15	0.21	0.08	0.10	1.02	0.09
β-Pinene	0.37	0.20	0.65	0.72	0.39	0.25	0.74	0.62
1-Octen-3-ol	0.04	0.10			0.01	0.01	0.02	0.01
3-Octanone			0.01	0.01				
β-Mircene	2.70	3.90	0.33	0.39	1.92	1.51	2.85	1.67
3-Octanol			0.10	0.08	0.04	0.07	0.04	0.02
Pseudolimonene	0.03	0.02	0.03	0.04				
α-Phellandrene	0.05	0.04			0.34	0.29	0.88	0.27
δ-3-Carene					0.16	0.08	0.01	0.06
α-Terpinene	0.40	0.30	0.01	0.02	2.54	2.81	0.11	2.82
p-Cymene	1.17	0.87	0.04	0.06	9.25	9.67	0.41	10.43
dl-Limonene	1.41	0.84	3.35	2.37	0.72	0.59	7.05	0.61
Eucalyptol	40.22	60.94	0.07	0.08	0.01	0.01		
β-Ocimene, *trans-*	0.01	0.02	0.02	0.02	0.01	0.01	11.45	1.98
β-Ocimene, *cis-*	0.67	0.53	0.02	0.03	0.07	0.06	5.05	0.58
γ-Terpinene					14.95	17.71	0.18	14.57
Sabinene hydrate, *cis-*					0.06	0.06	0.08	0.09
Terpinolene	0.16	0.11	0.02	0.03	0.14	0.10	0.26	0.10
p-Cymenene	0.04	0.03			0.03	0.04	0.11	0.03
Linalool	0.13	0.15	0.48	0.25	1.25	2.38	7.37	1.19
Thujone, *cis-*	4.26	4.89	0.02	0.01	0.03		0.11	
Thujone, *trans-*	1.71	1.29			0.02	0.02		
Chrysanthenone					0.06	0.05	0.03	0.03
α-Campholenal							0.19	0.01
trans-Pinocarveol	0.21	0.12						
Sabinol, *cis-*							0.35	0.05
Verbenol, *cis-*							0.76	0.03
Camphor	14.88	1.89						
trans-3-Caren-2-ol							0.16	0.01
Menthone			0.38	0.56				
Pinocarvone							0.10	0.01
δ-Terpineol	0.76	0.81	12.22	17.20				
Borneol	1.59				0.60	0.74	1.20	0.35
Isopulegone			1.51	1.65				
Terpinene-4-ol	1.33	0.73	0.08	0.08	1.17	0.95	0.37	0.86
p-Cymen-8-ol					0.03	0.06	0.17	0.10
Hexyl butanoate					0.04	0.03		
α-Terpineol	2.61	2.71	0.08	0.06	0.14	0.11	14.92	0.29
Decanal			0.03	0.03	0.02	0.01		
Verbenone			0.04	0.03	0.01	0.01	0.15	0.01
Carveol, *trans-*							0.11	0.01
Nerol	0.05	0.01			0.04	0.03		
Thymol, methyl ether							0.01	1.65
Carvacrol, methyl ether							0.01	3.87
Pulegone			79.48	75.1				
Cumin aldehyde							0.08	0.02
Z-Citral	0.04							
Bornyl acetate	0.06						0.09	
Thymol	0.02	0.52			31.12	35.66	0.11	46.10
Carvacrol	0.02	0.24			26.01	17.44	0.10	2.17
Piperitenone			0.13	0.11				
thymol acetate	0.45	0.13			0.35	0.77		0.55
Carvacrol acetate					0.26	0.41		
α-Copaene							0.69	0.04
β-Elemene							0.04	0.02
α-Gurjunene					0.04	0.02	0.06	0.10
Caryophyllene, *trans-*	1.78	1.72	0.06	0.04	2.42	2.29	0.31	0.90
α-Bergamotene, *trans-*					0.02	0.02	0.11	0.04
Aromadendrene	0.25	0.22			0.04	0.03	0.58	0.03
α-Humulene	0.57	0.66			0.03	0.01	0.10	0.03
β-Santalene					0.08	0.07	0.02	0.03
Alloaromadendrene					0.04	0.02	0.04	0.01
Germacrene D			0.08	0.06			0.31	0.59
Guaia-1(10),11-diene	0.08	0.06			0.04	0.03	0.07	0.02
β-Guaiene, *trans*-	0.10	0.04			0.19	0.29	1.85	0.09
β-Bisabolene					0.29	0.28		3.53
β-Curcumene							0.63	
α-Muurolene	0.05	0.04					0.07	0.04
Calamenene, *cis*-	0.11	0.10			0.03	0.04	0.08	0.04
Sesquiphellandrene							0.03	0.04
α-Bisabolene, (E)-					0.22	0.24		0.10
Sesquisabinene hydrate, *cis*-							2.79	0.06
trans-Sesquisabinene hydrate							0.15	0.01
(-)-Spathulenol							0.61	0.03

**Table 6:** List of the identified compounds in the essential oils extracts and the identification method (IM) used: MS: Mass Spectrum search against Nist and Wiley database; RI: experimental and theoretical Retention Index comparison; RI ref: Retention Index reference; RT: Retention Time.

Compound Name	RT	RI	RI Ref	IM
Methyl 3(Z)-Hexenyl Ether	4.341	823		MS
cis-Salvene	4.783	845		MS
3-Hexen-1-ol, *trans-*	4.833	847	856	MS/RI
trans-Salvene	4.971	854		MS
2-Hexen-1-ol, *trans-*	5.033	857		MS
1-Hexanol	5.112	861	870	MS/RI
2-α-Pinene	6.363	918		MS
Tricyclene	6.421	923	923	MS/RI
α-Thujene	6.492	925	927	MS/RI
α-Pinene, (-)-	6.692	933	936	MS/RI
2,4(10)-thujadien	6.938	943	943	MS/RI
Camphene	7.117	950	950	MS/RI
verbenene	7.209	951	963	MS/RI
Sabinene	7.755	972	973	MS/RI
β-Pinene	7.897	977	977	MS/RI
1-Octen-3-ol	7.98	980	980	MS/RI
3-Octanone	8.039	983	984	MS/RI
β-Mircene	8.118	988	989	MS/RI
3-Octanol	8.351	995	993	MS/RI
Pseudolimonene	8.539	1002	992	MS/RI
α-Phellandrene	8.597	1004	1004	MS/RI
δ-3-Carene	8.743	1009	1005	MS/RI
α-Terpinene	8.898	1017	1017	MS/RI
p-Cymene	9.102	1024	1024	MS/RI
dl-Limonene	9.252	1029	1029	MS/RI
Eucalyptol	9.348	1032	1032	MS/RI
β-Ocimene, *trans-*	9.698	1035	1035	MS/RI
β-Ocimene, *cis-*	9.769	1044	1041	MS/RI
γ-Terpinene	10.052	1058	1060	MS/RI
Sabinene hydrate, *cis-*	10.448	1069	1066	MS/RI
Terpinolene	10.832	1085	1087	MS/RI
p-Cymenene	10.944	1087	1088	MS/RI
Linalool	11.195	1098	1099	MS/RI
Thujone, *cis-*	11.42	1104	1105	MS/RI
Thujone, *trans-*	11.741	1116	1115	MS/RI
Chrysanthenone	11.991	1124	1124	MS/RI
α-Campholenal	12.016	1125	1124	MS/RI
trans-Pinocarveol	12.416	1140	1140	MS/RI
Sabinol, *cis*-	12.454	1142	1142	MS/RI
Verbenol, *cis*	12.558	1144	1144	MS/RI
Camphor	12.595	1146	1143	MS/RI
trans-3-Caren-2-ol	12.695	1149		MS
Menthone	12.87	1155	1150	MS/RI
Pinocarvone	13.037	1161	1161	MS/RI
δ-Terpineol	13.221	1165	1165	MS/RI
Borneol	13.287	1170	1166	MS/RI
Isopulegone	13.417	1175	1176	MS/RI
Terpinene-4-ol	13.529	1178	1177	MS/RI
p-Cymen-8-ol	13.733	1181	1184	MS/RI
Hexyl butanoate	13.867	1191	1191	MS/RI
α-Terpineol	13.925	1195	1190	MS/RI
Decanal	14.25	1204	1200	MS/RI
Verbenone	14.346	1208	1206	MS/RI
Carveol, *trans-*	14.605	1215	1217	MS/RI
Nerol	14.717	1221	1229	MS/RI
Thymol, methyl ether	14.901	1231	1234	MS/RI
Carvacrol, methyl ether	15.113	1236	1243	MS/RI
Pulegone	15.251	1239	1237	MS/RI
Cumin aldehyde	15.288	1242	1238	MS/RI
Z-Citral	15.893	1264		MS
Bornyl acetate	16.389	1283	1283	MS/RI
Thymol	16.535	1290	1290	MS/RI
Carvacrol	16.718	1299	1300	MS/RI
Piperitenone	17.844	1337	1340	MS/RI
thymol acetate	18.027	1345	1354	MS/RI
Carvacrol acetate	18.519	1363	1356	MS/RI
α-Copaene	18.823	1378	1376	MS/RI
β-Elemene	19.207	1391	1390	MS/RI
α-Gurjunene	19.695	1409	1408	MS/RI
Caryophyllene, (E)-	19.982	1421	1420	MS/RI
α-Bergamotene, *trans-*	20.328	1423	1434	MS/RI
Aromadendrene	20.453	1440	1441	MS/RI
α-Humulene	20.874	1452	1453	MS/RI
β-Santalene	20.916	1458	1458	MS/RI
Alloaromadendrene	21.029	1463	1460	MS/RI
Germacrene D isomer	21.387	1477		MS
Germacrene D	21.521	1483	1481	MS/RI
Guaia-1(10),11-diene	21.746	1492		MS
β-Guaiene, *trans-*	21.871	1498	1499	MS/RI
β-Bisabolene	22.142	1509	1508	MS/RI
β-Curcumene	22.158	1510	1513	MS/RI
α-Muurolene	22.254	1514	1498	MS/RI
Calamenene, *cis-*	22.421	1522	1522	MS/RI
Sesquiphellandrene	22.5	1525	1523	MS/RI
α-Bisabolene, (E)-	22.9	1543	1540	MS/RI
Sesquisabinene hydrate, *cis-*	22.955	1546	1541	MS/RI
Sesquisabinene hydrate, *trans-*	23.201	1557		MS
Caryophyllene oxide	23.313	1562	1580	MS/RI
(-)-Spathulenol	23.743	1581	1576	MS/RI
Aromadendrene oxide-	23.847	1585		MS
a-Humulene oxide	24.097	1596	1601	MS/RI

## Data Availability

Not applicable.
